# Correction: Weimer et al. Localisation-Dependent Variations in Articular Cartilage ECM: Implications for Tissue Engineering and Cartilage Repair. *Int. J. Mol. Sci.* 2025, *26*, 9331

**DOI:** 10.3390/ijms27073252

**Published:** 2026-04-03

**Authors:** Laura Weimer, Luisa M. Schmidt, Gerhard Sengle, Marcus Krüger, Alan M. Smith, Ilona Brändlin, Frank Zaucke

**Affiliations:** 1 Faculty 2: Computer Science and Engineering, Frankfurt University of Applied Sciences, 60318 Frankfurt, Germany; laura.weimer@fra-uas.de (L.W.); ilona.braendlin@fra-uas.de (I.B.); 2 Department of Pharmacy, School of Applied Sciences, University of Huddersfield, Huddersfield HD1 3DH, UK; a.m.smith@hud.ac.uk; 3 Institute for Genetics, Cologne Excellence Cluster on Cellular Stress Responses in Ageing-Associated Diseases (CECAD), and Center for Molecular Medicine Cologne (CMMC), University of Cologne, 50931 Cologne, Germany; luisa.schmidt@cpr.ku.dk (L.M.S.); marcus.krueger@uni-koeln.de (M.K.); 4 Novo Nordisk Foundation Center for Protein Research, Department of Cellular and Molecular Medicine, Faculty of Health and Medical Sciences, University of Copenhagen, 1172 Copenhagen, Denmark; 5 Department of Pediatrics and Adolescent Medicine, Faculty of Medicine and University Hospital Cologne, Center for Biochemistry, Center for Molecular Medicine Cologne (CMMC), Cologne Center for Musculoskeletal Biomechanics (CCMB), and Cologne Excellence Cluster on Cellular Stress Responses in Ageing-Associated Diseases (CECAD), University of Cologne, 50931 Cologne, Germany; gsengle@uni-koeln.de; 6 Dr. Rolf M. Schwiete Osteoarthritis Research Unit, Department of Trauma Surgery and Orthopedics, University Hospital, Goethe University, 60528 Frankfurt, Germany

**Missing Institutional Review Board Statement and Informed Consent Statement**

In the original publication [1], Institutional Review Board Statement and Informed Consent Statement were not included. The correct Missing Institutional Review Board Statement and Informed Consent Statement are as below. The authors state that the scientific conclusions are unaffected. This correction was approved by the Academic Editor. The original publication has also been updated.

**Institutional Review Board Statement:** Knee joints were obtained from pigs that were slaughtered by a local butcher for food production purposes and therefore did not require ethical approval.
**Informed Consent Statement:** Not applicable.
