# Supplementary material for: Localisation-Dependent Variations in Articular Cartilage ECM: Implications for Tissue Engineering and Cartilage Repair
Source: Int J Mol Sci. 2025 Sep 24;26(19):9331. doi: 10.3390/ijms26199331 (PMC12524475; doi:10.3390/ijms26199331)
Supplement: Supplementary file 1 [file ijms-26-09331-s001.zip › ijms-3853748-supplementary/Supplement S2 - homology human vs porcine protein sequences.pdf]

Homology proteins human vs porcine

FASTA data from UniProt database (https://www.uniprot.org/)
Protein BLAST NIH datzabase (https://blast.ncbi.nlm.nih.gov/Blast.cgi) - BLASTp

Decorin

Homo sapiens FASTA
MKATIILLLLAQVSWAGPFQQRGLFDFMLEDEASGIGPEVPDDRDFEPSLGPVCPFRCQCHLRVVQC
SDLGLDKVPKDLPPDTTLLDLQNNKITEIKDGDFKNLKNLHALILVNNKISKVSPGAFTPLVKLERLY
LSKNQLKELPEKMPKTLQELRAHENEITKVRKVTFNGLNQMIVIELGTNPLKSSGIENGAFQGMKKL
SYIRIADTNITSIPQGLPPSLTELHLDGNKISRVDAASLKGLNNLAKLGLSFNSISAVDNGSLANTPHL
RELHLDNNKLTRVPGGLAEHKYIQVVYLHNNNISVVGSSDFCPPGHNTKKASYSGVSLFSNPVQYW
EIQPSTFRCVYVRSAIQLGNYK
Sus scrofa FASTA
MKATIVFLLLAQVSWAGPFQQKGLFDFMLEDEASGIGPEDRFPEVPELEPLGPMCPFRCQCHLRVV
QCSDLGLDKVPKDLPPDTALLDLQNNKITEIKDGDFKNLKNLHTLILINNKISKISPGAFAPLVKLER
LYLSKNQLKELPEKMPKTLQELRVHENEITKVRKAVFNGLNQMIVVELGTNPLKSSGIENGAFQGM
KKLSYIRIADTNITTIPQGLPPSLTELHLDGNKISKVDAASLKGLNNLAKLGLGFNSISTVDNGSLANT
PHLRELHLNNNKLNKVPGGLAEHKYIQVVYLHNNNISAVGSNDFCPPGYNTKKASYSGVSLFSNP
VQYWEIQPSTFRCVYVRSAIQLGNYK

Homology: 91.14 %

Matrilin-3

Homo sapiens FASTA
MPRPAPARRLPGLLLLLWPLLLLPSAAPDPVARPGFRRLETRGPGGSPGRRPSPAAPDGAPASGTSEP
GRARGAGVCKSRPLDLVFIIDSSRSVRPLEFTKVKTFVSRIIDTLDIGPADTRVAVVNYASTVKIEFQLQ
AYTDKQSLKQAVGRITPLSTGTMSGLAIQTAMDEAFTVEAGAREPSSNIPKVAIIVTDGRPQDQVNE
VAARAQASGIELYAVGVDRADMASLKMMASEPLEEHVFYVETYGVIEKLSSRFQETFCALDPCVLG
THQCQHVCISDGEGKHHCECSQGYTLNADKKTCSALDRCALNTHGCEHICVNDRSGSYHCECYE
GYTLNEDRKTCSAQDKCALGTHGCQHICVNDRTGSHHCECYEGYTLNADKKTCSVRDKCALGSH
GCQHICVSDGAASYHCDCYPGYTLNEDKKTCSATEEARRLVSTEDACGCEATLAFQDKVSSYLQRL
NTKLDDILEKLKINEYGQIHR
Sus scrofa FASTA
QDQARARYLPALLLLLWPLLLLPAAADPSPLARPGFRRLGTRGPGGSPGRRPAAAAPTHAPYSGA
GQPGRARGAGICKSRPLDLVFIIDSSRSVRPLEFTKVKTFVSRIIDNLDIGAEDTRVAVVNYASTVKIEF
HLQTHSDKQALKRAVARIAPLSTGTMSGLAIQTAMDKAFTVEAGARGPNSNIPKVAIIVTDGRPQD
QVNEVAARARASGIELYAVGVDRADMESLRLMASEPLDEHVFYVETYGVIEKLSSRFQETFCALDPC
LLGTHQCQHVCISDGEGKHHCECSQGYSLNADKKTCSAIDKCALNTHGCEHICVNDRTGSYHCEC
YEGYTLNEDRKTCSARDQCALGTHGCQHICVNDGAGSHHCECYEGYILNEDKKTCSVRNKCALGS
HGCQHICVNDGAGAYHCECYTGYTLNEDKKTCSAIEEARRLISTEDACACEATLAFQDKVSSYLQR
LNSKLDDILEKLQANEYGQIHR

Homology: 88.24 %

Fibrillin-1

Homo sapiens FASTA

MRRGRLLEIALGFTVLLASYTSHGADANLEAGNVKETRASRAKRRGGGGHDALKGPNVCGSRYN
AYCCPGWKTLPGGNQCIVPICRHSCGDGFCSRPNMCTCPSGQIAPSCGSRSIQHCNIRCMNGGSCS
DDHCLCQKGYIGTHCGQPVCESGCLNGGRCVAPNRCACTYGFTGPQCERDYRTGPCFTVISNQMC
QGQLSGIVCTKTLCCATVGRAWGHPCEMCPAQPHPCRRGFIPNIRTGACQDVDECQAIPGLCQGG
NCINTVGSFECKCPAGHKLNEVSQKCEDIDECSTIPGICEGGECTNTVSSYFCKCPPGFYTSPDGTRCI
DVRPGYCYTALTNGRCSNQLPQSITKMQCCCDAGRCWSPGVTVAPEMCPIRATEDFNKLCSVPMVI
PGRPEYPPPPLGPIPPVLPVPPGFPPGPQIPVPRPPVEYLYPSREPPRVLPVNVTDYCQLVRYLCQNGR
CIPTPGSCRCECNKGFQLDLRGECIDVDECEKNPCAGGECINNQGSYTCQCRAGYQSTLTRTECRDI
DECLQNGRICNNGRCINTDGSFHCVCNAGFHVTRDGKNCEDMDECSIRNMCLNGMCINEDGSFK
CICKPGFQLASDGRYCKDINECETPGICMNGRCVNTDGSYRCECFPGLAVGLDGRVCVDTHMRSTC
YGGYKRGQCIKPLFGAVTKSECCCASTEYAFGEPCQPCPAQNSAEYQALCSSGPGMTSAGSDINECA
LDPDICPNGICENLRGTYKCICNSGYEVDSTGKNCVDINECVLNSLLCDNGQCRNTPGSFVCTCPKG
FIYKPDLKTCEDIDECESSPCINGVCKNSPGSFICECSSESTLDPTKTICIETIKGTCWQTVIDGRCEININ
GATLKSQCCSSLGAAWGSPCTLCQVDPICGKGYSRIKGTQCEDIDECEVFPGVCKNGLCVNTRGSFK
CQCPSGMTLDATGRICLDIRLETCFLRYEDEECTLPIAGRHRMDACCCSVGAAWGTEECEECPMRN
TPEYEELCPRGPGFATKEITNGKPFFKDINECKMIPSLCTHGKCRNTIGSFKCRCDSGFALDSEERNCT
DIDECRISPDLCGRGQCVNTPGDFECKCDEGYESGFMMMKNCMDIDECQRDPLLCRGGVCHNTEG
SYRCECPPGHQLSPNISACIDINECELSAHLCPNGRCVNLIGKYQCACNPGYHSTPDRLFCVDIDECS
IMNGGCETFCTNSEGSYECSCQPGFALMPDQRSCTDIDECEDNPNICDGGQCTNIPGEYRCLCYDGF
MASEDMKTCVDVNECDLNPNICLSGTCENTKGSFICHCDMGYSGKKGKTGCTDINECEIGAHNCG
KHAVCTNTAGSFKCSCSPGWIGDGIKCTDLDECSNGTHMCSQHADCKNTMGSYRCLCKEGYTGD
GFTCTDLDECSENLNLCGNGQCLNAPGGYRCECDMGFVPSADGKACEDIDECSLPNICVFGTCHN
LPGLFRCECEIGYELDRSGGNCTDVNECLDPTTCISGNCVNTPGSYICDCPPDFELNPTRVGCVDTRS
GNCYLDIRPRGDNGDTACSNEIGVGVSKASCCCSLGKAWGTPCEMCPAVNTSEYKILCPGGEGFRP
NPITVILEDIDECQELPGLCQGGKCINTFGSFQCRCPTGYYLNEDTRVCDDVNECETPGICGPGTCYN
TVGNYTCICPPDYMQVNGGNNCMDMRRSLCYRNYYADNQTCDGELLFNMTKKMCCCSYNIGRA
WNKPCEQCPIPSTDEFATLCGSQRPGFVIDIYTGLPVDIDECREIPGVCENGVCINMVGSFRCECPVG
FFYNDKLLVCEDIDECQNGPVCQRNAECINTAGSYRCDCKPGYRFTSTGQCNDRNECQEIPNICSH
GQCIDTVGSFYCLCHTGFKTNDDQTMCLDINECERDACGNGTCRNTIGSFNCRCNHGFILSHNND
CIDVDECASGNGNLCRNGQCINTVGSFQCQCNEGYEVAPDGRTCVDINECLLEPRKCAPGTCQNL
DGSYRCICPPGYSLQNEKCEDIDECVEEPEICALGTCSNTEGSFKCLCPEGFSLSSSGRRCQDLRMSYC
YAKFEGGKCSSPKSRNHSKQECCCALKGEGWGDPCELCPTEPDEAFRQICPYGSGIIVGPDDSAVD
MDECKEPDVCKHGQCINTDGSYRCECPFGYILAGNECVDTDECSVGNPCGNGTCKNVIGGFECTC
EEGFEPGPMMTCEDINECAQNPLLCAFRCVNTYGSYECKCPVGYVLREDRRMCKDEDECEEGKHD
CTEKQMECKNLIGTYMCICGPGYQRRPDGEGCVDENECQTKPGICENGRCLNTRGSYTCECNDGFT
ASPNQDECLDNREGYCFTEVLQNMCQIGSSNRNPVTKSECCCDGGRGWGPHCEICPFQGTVAFKK
LCPHGRGFMTNGADIDECKVIHDVCRNGECVNDRGSYHCICKTGYTPDITGTSCVDLNECNQAPK
PCNFICKNTEGSYQCSCPKGYILQEDGRSCKDLDECATKQHNCQFLCVNTIGGFTCKCPPGFTQHH
TSCIDNNECTSDINLCGSKGICQNTPGSFTCECQRGFSLDQTGSSCEDVDECEGNHRCQHGCQNIIG
GYRCSCPQGYLQHYQWNQCVDENECLSAHICGGASCHNTLGSYKCMCPAGFQYEQFSGGCQDIN
ECGSAQAPCSYGSSNTEGGYLCGCPPGYFRIGQGHCVSGMGMGRGNPEPPVSGEMDDNSLSPEAC
YECKINGYPKRGRKRRSTNETDASNIEDQSETEANVSLASWDVEKTAIFAFNISHVSNKVRILELLPA

LTTLTNHNRYLIESGNEDGFFKINQKEGISYLHFTKKKPVAGTYSLQISSTPLYKKKELNQLEDKYDK
DYLSGELGDNLKMKIQVLLH

Sus scrofa FASTA

MRRGRLLEVALGFTVLLASYTSHRAEANLEAGNGKETRASRAKRRGGGGHDALKGPNVCGSRYN
AYCCPGWKTLPGGNQCIVPICRHSCGDGFCSRPNMCTCPSGQIAPSCGSRSIQHCNIRCMNGGSCS
DDHCLCQKGYIGTHCGQPVCESGCLNGGRCVAPNRCACTYGFTGPQCERDYRTGPCFTVVSNQM
CQGQLSGIVCTKTLCCATVGRAWGHPCEMCPAQPHPCRRGFIPNIRTGACQDVDECQAIPGLCQG
GNCINTVGSFECKCPAGHKFNEVSQKCEDIDECSTIPGICDGGECTNTVSSYFCKCPPGFYTSPDGTR
CIDVRPGYCYTALTNGRCSNQLPQSITKMQCCCDVGRCWSPGVTVTPEMCPIRATEDFNKLCSVPM
VVPERPGYPSPPLGPIPPVHPVPPGFPPGPQIPVPRPPVEYPYPSREPPRVLPVNVTDYCQLFRYLCHN
GRCIPTPGSYRCECNKGFQLDLRGECIDVDECEKNPCAGGECINNQGSYTCQCRPGYQSTLTRTECR
DIDECLQNGRICNNGRCINTDGSFHCVCNAGFHVTRDGKNCEDMDECSIRNMCLNGMCINEDGSF
KCICKPGFQLASDGRYCKDINECETSGICMNGRCVNTDGSYRCECFPGLAVGLDGRVCVDTHMRST
CYGGYKRGQCVKPLFGAVTKSECCCASTEYAFGEPCQPCPSQNSAEYQALCSSGPGMTSAGSDINE
CALDPDICPNGICENLRGTYKCICNSGYEVDSTGKNCVDINECVLNSLLCDNGQCRNTPGSFVCTCP
KGFIYKPDLKTCEDIDECESSPCINGVCKNSPGSFICECSSESTLDPTKTICIETIKGTCWQTIIDGRCEIN
INGATLKSQCCSSLGAAWGSPCTPCQVDPICGKGYSRIKGTQCEDIDECEVFPGVCKNGLCVNSKGS
FKCQCPNGMTLDATGRICLDIRLETCFLRYEDEECTLPVVGRHRMDACCCSVGAAWGTEECEECPP
RNTPEYEELCPRGPGFATKEITNGKPFFKDINECKMIPNLCTHGKCRNTIGSFKCRCDSGFALDSEER
NCIDIDECRISPDLCGRGQCVNTPGDFECKCDEGYESGFMMMKNCMDIDECQRDPLLCRGGVCLN
TEGSYRCECPSGHQMSPNISACIDINECELSAHLCPHGRCVNLIGKYQRARNPGYHSTPDRLFCVDI
DECSIMNGGCETFCTNSEGSYECSCQPGFALMPDQRSCTDIDECEDNPNICDGGQCTNIPGEYRCLC
YDGFMASEDMKTCVDVNECDLNPNICLSGTCENTKGSFICHCDMGYSGKKGKTGCTDINECEIGA
HNCDRHAVCTNTAGSFNCSCSPGWIGDGIKCTDLDECSNGTHMCSQHADCKNTMGSYRCLCKEG
YTGDGFTCADLDECSENVKLCGNVQCLYAPGGYHCEYDMGFVPSADRKSCVDSDECSLPNICVFG
TCHNLPGLFRCECEIGYELDRSGGNCTDVNECLEPPTCISGNCVNTPGSYTCVCPPDFELNPTRVGC
VDTRSGNCYLDVRPRGDNGDTACSNEIGVGVSKASCCCSLGKAWGTPCEQCPPVNTSEYKILCPGG
EGFRPNPITVILEDIDECQELPGLCQGGKCINTFGSFQCRCPTGYYLNEDTRVCDDVNECETPGICGP
GTCYNTVGNYTCICPPDYMQVNGGNNCMDMRRSLCYRNYYADNQTCDGELLFNMTKKMCCCSY
NIGRAWNKPCEQCPIPSTDEFATLCGSQRPGFVIDIYTGLPVDIDECREIPGVCENGVCINMVGSFRC
ECPVGFFYNDKLLVCEDIDECQNGPVCQRNAECINTAGSYRCDCKPGYRFTSTGQCNDRNECQEIP
NICSHGQCIDTVGSFYCLCHTGFKTNADQTMCLDINECERDACGNGTCRNTIGSFNCRCNHGFILS
HNNDCIDVDECATGNGNLCRNGQCINTVGSFQCQCNEGYEVAPDGRTCVDINECLLEPGKCAPG
TCQNLDGSYRCICPPGYSLQNDKCEDIDECVEEPEICALGTCSNTEGSFKCLCPDGFSLSSTGRRCQD
LRMSYCYAKFEGGKCSSPKSRNHSKQECCCALKGEGWGDPCELCPTEPDEAFRQICPYGSGIIVGPD
DSAVDMDECKEPDVCKHGQCINTDGSYRCECPFGYILEGNECVDTDECSVGNPCGNGTCKNVIGG
FECTCEEGFEPGPMMTCEDINECAQNPLLCAFRCVNTYGSYECKCPTGYVLREDRRMCKDEDECEE
GKHDCAEKQMECKNLIGMYICICGPGYQRRPDGEGCVDENECQTKPGICENGRCLNTRGSYTCEC
NDGFTASPTQDECLDNREGYCFTEVLQNMCQIGSSNRNPVTKSECCCDGGRGWGPHCEICPFQGT
VAFKKLCPHGRGFMTNGADIDECKVIHDVCRNGECINDRGSYHCICKTGYTPDITGTACVDLNEC
NQAPKPCNFICKNTEGSYQCSCPKGYILQEDGRSCKDLDECATKQHNCQFLCVNTIGSFACKCPPG
FTQHHTACIDNNECTSDINLCGAKGICQNTPGSFTCECQRGFSLDQSGASCEDVDECEGNHRCQH
GCQNIIGGYRCSCPQGYLQHYQWNQCVDENECLSAHICGGASCHNTLGSYKCMCPAGFQYEQFSG
GCQDINECGSSQAPCSYGCSNTEGGYLCGCPPGYFRIGQGHCVSGMGMGRGSPEPPASGEMDDNS
LSPEACYECKINGYPKRGRKRRSTNETDAFNIEDQPETESNVSLASWDVEKTAVFAFNISHISNKVRIL

ELLPALTTLTNHNRYLIESGNENGFFKINQKEGISYLHFTKKKPVAGTYSLQISSTPLYKKKELNQLED
KYDKDYLSGELGDNLKMKIQILLH

Homology: 96.87 %

Collagen type II

Homo sapiens FASTA

MIRLGAPQTLVLLTLLVAAVLRCQGQDVQEAGSCVQDGQRYNDKDVWKPEPCRICVCDTGTVLC
DDIICEDVKDCLSPEIPFGECCPICPTDLATASGQPGPKGQKGEPGDIKDIVGPKGPPGPQGPAGEQG
PRGDRGDKGEKGAPGPRGRDGEPGTPGNPGPPGPPGPPGPPGLGGNFAAQMAGGFDEKAGGAQL
GVMQGPMGPMGPRGPPGPAGAPGPQGFQGNPGEPGEPGVSGPMGPRGPPGPPGKPGDDGEAGK
PGKAGERGPPGPQGARGFPGTPGLPGVKGHRGYPGLDGAKGEAGAPGVKGESGSPGENGSPGPM
GPRGLPGERGRTGPAGAAGARGNDGQPGPAGPPGPVGPAGGPGFPGAPGAKGEAGPTGARGPEG
AQGPRGEPGTPGSPGPAGASGNPGTDGIPGAKGSAGAPGIAGAPGFPGPRGPPGPQGATGPLGPKG
QTGEPGIAGFKGEQGPKGEPGPAGPQGAPGPAGEEGKRGARGEPGGVGPIGPPGERGAPGNRGFP
GQDGLAGPKGAPGERGPSGLAGPKGANGDPGRPGEPGLPGARGLTGRPGDAGPQGKVGPSGAPG
EDGRPGPPGPQGARGQPGVMGFPGPKGANGEPGKAGEKGLPGAPGLRGLPGKDGETGAAGPPGP
AGPAGERGEQGAPGPSGFQGLPGPPGPPGEGGKPGDQGVPGEAGAPGLVGPRGERGFPGERGSPG
AQGLQGPRGLPGTPGTDGPKGASGPAGPPGAQGPPGLQGMPGERGAAGIAGPKGDRGDVGEKGP
EGAPGKDGGRGLTGPIGPPGPAGANGEKGEVGPPGPAGSAGARGAPGERGETGPPGPAGFAGPPG
ADGQPGAKGEQGEAGQKGDAGAPGPQGPSGAPGPQGPTGVTGPKGARGAQGPPGATGFPGAAG
RVGPPGSNGNPGPPGPPGPSGKDGPKGARGDSGPPGRAGEPGLQGPAGPPGEKGEPGDDGPSGAE
GPPGPQGLAGQRGIVGLPGQRGERGFPGLPGPSGEPGKQGAPGASGDRGPPGPVGPPGLTGPAGEP
GREGSPGADGPPGRDGAAGVKGDRGETGAVGAPGAPGPPGSPGPAGPTGKQGDRGEAGAQGPM
GPSGPAGARGIQGPQGPRGDKGEAGEPGERGLKGHRGFTGLQGLPGPPGPSGDQGASGPAGPSGP
RGPPGPVGPSGKDGANGIPGPIGPPGPRGRSGETGPAGPPGNPGPPGPPGPPGPGIDMSAFAGLGPR
EKGPDPLQYMRADQAAGGLRQHDAEVDATLKSLNNQIESIRSPEGSRKNPARTCRDLKLCHPEWK
SGDYWIDPNQGCTLDAMKVFCNMETGETCVYPNPANVPKKNWWSSKSKEKKHIWFGETINGGFH
FSYGDDNLAPNTANVQMTFLRLLSTEGSQNITYHCKNSIAYLDEAAGNLKKALLIQGSNDVEIRAE
GNSRFTYTALKDGCTKHTGKWGKTVIEYRSQKTSRLPIIDIAPMDIGGPEQEFGVDIGPVCFL

Sus scrofa FASTA

MIRLGAPQTLVLLTLLVAAVLRCHGQDVQKAGSCVQDGQRYNDKDVWKPEPCRICVCDTGTVLC
DDIICEDLKDCLSPETPFGECCPICSTDLATASGQLGPKGQKGEPGDIKDIVGPKGPPGPQGPAGEQG
PRGDRGDKGEKGAPGPRGRDGEPGTPGNPGPPGPPGPPGPPGLGGNFAAQMAGGFDEKAGGAQ
MGVMQGPMGPMGPRGPPGPAGAPGPQGFQGNPGEPGEPGVSGPMGPRGPPGPPGKPGDDGEAG
KPGKSGERGPPGPQGARGFPGTPGLPGVKGHRGYPGLDGAKGEAGAPGVKGESGSPGENGSPGPM
GPRGLPGERGRTGPAGAAGARGNDGQPGPAGPPGPVGPAGGPGFPGAPGAKGEAGPTGARGPEG
AQGPRGEPGNPGSPGPAGASGNPGTDGIPGAKGSAGAPGIAGAPGFPGPRGPPGPQGATGPLGPK
GQTGEPGIAGFKGEQGPKGEPGPAGPQGAPGPAGEEGKRGARGEPGGAGPAGPPGERGAPGNRG
FPGQDGLAGPKGAPGERGPSGLAGPKGANGDPGRPGEPGLPGARGLTGRPGDAGPQGKVGPSGA
PGEDGRPGPPGPQGARGQPGVMGFPGPKGANGEPGKAGEKGLPGAPGLRGLPGKDGETGAAGPP
GPAGPAGERGEQGAPGPSGFQGLPGPPGAPGEGGKPGDQGVPGEAGAPGVGPRGERGFPGERGSP
GSQGLQGPRGLPGTPGTDGPKGASGPAGPPGAQGPPGLQGMPGERGAAGIAGPKGDRGDVGEKG
PEGAPGKDGGRGLTGPIGPPGPAGANGEKGEVGPPGPAGTAGARGAPGERGETGPPGPAGFAGPP
GADGQPGAKGEQGEAGQKGDAGAPGPQGPSGAPGPQGPTGVTGPKGARGAQGPPGATGFPGAA

GRVGPPGSNGNPGPPGPPGPSGKDGPKGARGDSGPPGRAGDPGLQGPAGPPGEKGEPGEDGPSGP
DGPPGPQGLAGQRGIVGLPGQRGERGFPGLPGPSGEPGKQGAPGASGDRGPPGPVGPPGLTGPSGE
PGREGSPGADGPPGRDGAAGVKGDRGETGAAGAPGAPGPPGSPGPAGPTGKQGDRGEAGAQGP
MGPAGPAGARGMPGPQGPRGDKGEAGEAGERGLKGHRGFTGLQGLPGPPGPSGDQGASGPAGPS
GPRGPPGPVGPSGKDGANGIPGPIGPPGPRGRSGETGPAGPPGTPGPPGPPGPPGPGIDMSAFAGLG
QREKGPDPLQYMRADEAAGNLRQHDAEVDATLKSLNNQIESIRSPEGSRKNPARTCRDLKLCHPE
WKSGDYWIDPNQGCTLDAMKVFCNMETGETCVYPSPASVPKKNWWSSKSKDKKHIWFGETINGG
FHFSYGDDNLAPNTANVQMTFLRLLSTEGSQNITYHCKNSIAYLDEAAGNLKKALLIQGSNDVEIR
AEGNSRFTYTVLKDGCTKHTGKWGQTMIEYRSQKTSRLPIIDIAPMDIGGPEQEFGVDIGPVCFL

<u>Homology: 97.65 %</u>

COMP

Homo sapiens FASTA

MVPDTACVLLLTLAALGASGQGQSPLGSDLGPQMLRELQETNAALQDVRELLRQQVREITFLKNTV
MECDACGMQQSVRTGLPSVRPLLHCAPGFCFPGVACIQTESGARCGPCPAGFTGNGSHCTDVNEC
NAHPCFPRVRCINTSPGFRCEACPPGYSGPTHQGVGLAFAKANKQVCTDINECETGQHNCVPNSV
CINTRGSFQCGPCQPGFVGDQASGCQRRAQRFCPDGSPSECHEHADCVLERDGSRSCVCAVGWAG
NGILCGRDTDLDGFPDEKLRCPERQCRKDNCVTVPNSGQEDVDRDGIGDACDPDADGDGVPNEK
DNCPLVRNPDQRNTDEDKWGDACDNCRSQKNDDQKDTDQDGRGDACDDDIDGDRIRNQADN
CPRVPNSDQKDSDGDGIGDACDNCPQKSNPDQADVDHDFVGDACDSDQDQDGDGHQDSRDNC
PTVPNSAQEDSDHDGQGDACDDDDDNDGVPDSRDNCRLVPNPGQEDADRDGVGDVCQDDFDA
DKVVDKIDVCPENAEVTLTDFRAFQTVVLDPEGDAQIDPNWVVLNQGREIVQTMNSDPGLAVGYT
AFNGVDFEGTFHVNTVTDDDYAGFIFGYQDSSSFYVVMWKQMEQTYWQANPFRAVAEPGIQLKA
VKSSTGPGEQLRNALWHTGDTESQVRLLWKDPRNVGWKDKKSYRWFLQHRPQVGYIRVRFYEGP
ELVADSNVVLDTTMRGGRLGVFCFSQENIIWANLRYRCNDTIPEDYETHQLRQA

Sus scrofa FASTA

MVLTVARVLLITLAALGASGQGQITLGADLGPQMLRELQETNAALQDVRELLRQQVKEITFLKNTV
MECDACGMQPARTPRLSVRPLSQCAPGFCFPGVACTETASGARCGPCPAGFTGNGSHCADVNEC
NAHPCFPRVRCINTSPGFRCEACPPGYSGPTHEGVGLAFAKANKQVCTDINECETGQHNCVPNSVC
VNTVGSFQCGPCQPGFVGDQASGCRRRSQRFCPDGTPSPCHEKADCVLERDGSRSCVCAVGWAGN
GILCGRDTDLDGFPDEKLRCSERQCRKDNCVTVPNSGQEDVDRDGIGDACDPDADGDGVLNEQD
NCPLVRNPDQRNADGDKWGDACDNCRSQKNDDQKDTDQDGRGDACDDDIDGDRIRNTVDNCP
RVPNSDQKDSDGDGIGDACDNCPQKSNADQRDVDHDFVGDACDSDQDKDGDGHQDSRDNCPT
VPNSAQQDSDHDGQGDACDDDDDNDGVPDSRDNCRLVPNPGQEDVDRDGVGDACQGDFDAD
KVVDKIDVCPENAEVTLTDFRAFQTVVLDPEGDAQIDPNWVVLNQGMEIVQTMNSDPGLAVGYT
AFNGVDFEGTFHVNTVTDDDYAGFIFGYQDSSSFYVVMWKQMEQTYWQANPFRAVAEPGIQLKA
VKSSTGPGEQLRNALWHTGDTASQVRLLWKDPRNVGWKDKTSYRWFLQHRPQVGYIRVRFYEGP
ELVADSNVVLDTTMRGGRLGVFCFSQENIIWANLRYRCNDTIPEDYEAQRLLQA

<u>Homology: 93.33 %</u>
